# Uveal Melanoma Metastasis to the Thyroid

**DOI:** 10.1155/2023/2118672

**Published:** 2023-08-16

**Authors:** Rokshana R. Thanadar, Uzma M. Siddiqui, Shi Bai, Runhua Hou

**Affiliations:** ^1^Endocrinology and Diabetes Center of Tidewater, 3205 Churchland Blvd, Chesapeake, VA 23321, USA; ^2^Endocrinology, Dartmouth-Hitchcock Clinic, 100 Hitchcock Way, Manchester, NH 03104, USA; ^3^Department of Pathology, University of Massachusetts Medical School, One Innovation Drive, Biotech Three, Worcester, MA 01605, USA; ^4^Thyroid Associates, Massachusetts General Hospital, 15 Parkman Street, Suite #730S, Boston, MA 02114, USA

## Abstract

**Background:**

Around 1.2 to 3.1% of thyroid malignancies are due to metastasis. Among them, cutaneous malignant melanomas constitute 4% of malignancy metastasized to the thyroid. Uveal melanoma is uncommon, and its metastasis to the thyroid has only rarely been reported. Hereby, we describe an unusual case of uveal melanoma metastasized to the thyroid and discuss the concept of correct diagnosis. *Case Report*. During a routine ophthalmological examination, an 86-year-old Caucasian female was found to have retinal detachment secondary to choroidal melanoma. She was treated with gamma knife which resulted in reduction of tumor size. Three months later, she was noted to have a goiter on physical examination. Follow-up thyroid ultrasonography demonstrated numerous vascularized nodules in both lobes. The fine needle aspiration (FNA) of the left dominant nodule was indeterminate the first time and nondiagnostic the second time. FNA of the right dominant nodule was nondiagnostic twice but showed malignant cells the third time. Subsequent immunohistochemistry staining of the FNA sample from the right thyroid nodule confirmed a profile consistent with malignant melanoma.

**Conclusion:**

It should be kept in mind that a thyroid nodule detected in a patient with a diagnosis of uveal melanoma can be metastasis and that uveal melanoma diagnosis should be taken into account for the examination of the thyroid tumors of these patients. It is important to employ immunohistochemical staining FNA examination of the patient with such tumors for markers associated with a patient's known malignancy to facilitate diagnosis.

## 1. Introduction

Although thyroid nodules are quite common, the rate of malignancy is only about 5–10%. Most of the thyroid cancers are due to primary thyroid malignancy and 1.2–3.1% of them are a result of metastasis from another cancer [1–4]. Renal cell cancer, lung cancer, colorectal cancer, and breast cancer are the most common types of cancer which metastasize to the thyroid [4, 5]. About 4% of metastases to the thyroid are due to melanoma [4, 5]. Uveal melanoma is an uncommon type of melanoma with a reported incidence of 5 cases per million and composes roughly 5% of all types of melanomas [6, 7]. Uveal melanoma arises from melanocytes in the uveal tract and accounts for 80% of all ocular melanomas [7, 8]. It is the most common type of ocular malignancy [9]. Uveal melanoma spreads through a hematogenous route, and liver is the most common site of metastases (82–93%) followed by lung, bone, skin, and other extrahepatic tissues [10–13]. Multiple sites of metastases were identified in 87% of patients [11]. There have been only two published cases of metastasis to the thyroid due to uveal melanoma [14, 15]. Hereby, we present the clinical presentation and diagnostic challenge of a unique case of thyroid metastasis from malignant uveal melanoma and review the relevant literature.

## 2. Case Presentation

**Figure 1:**
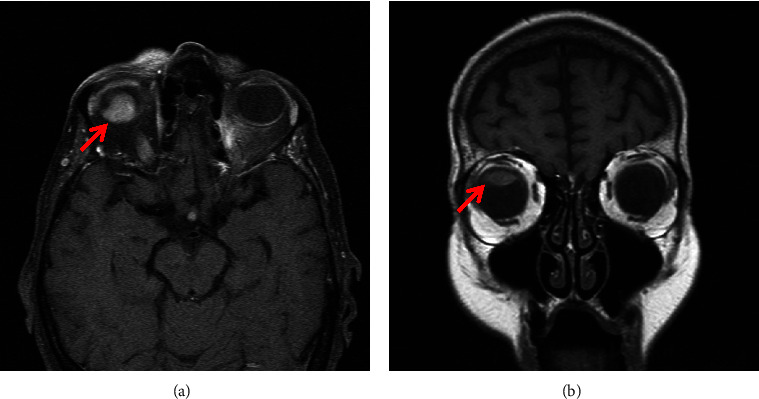
Transverse (a) and coronal (b) view of the orbit showing an 18 × 14 × 9 mm homogeneously enhancing mass (arrows) within the right globe.

An 86-year-old asymptomatic Caucasian female was found to have retinal detachment during a routine eye exam. Ophthalmoscopic examination in an outside hospital revealed superior serous retinal detachment with an underlying pigmented 20 × 22 mm large choroidal mass extending from the superior choroid to the superior ora serrata and temporally with subretinal fluid tracking into the macula. The apical height of the tumor was 9.6 mm on initial ultrasound. Subsequent magnetic resonance imaging (MRI) of the orbits identified an 18 × 14 × 9 mm homogeneously enhancing mass within the right globe, with its base at the posterior superior wall and extending exophytically into the vitreous of the posterior chamber with the suggestion of invasion through the superior wall of the orbit and into the surrounding orbital fat (Figures 1(a) and 1(b)). Given the patient's age and preference, she was treated with gamma knife radiosurgery which led to choroidal melanoma size reduction. The apical height of the tumor decreased to 6.98 mm on ultrasonography 6 months post radiation. The initial staging was not performed at the outside hospital for an unclear reason. Three months later, during a routine physical examination, she was noted to have thyromegaly and subsequent thyroid ultrasound revealed multiple thyroid nodules in both lobes. Her past medical history was significant for osteoporosis, mild cognitive impairment, hypertension, and chronic kidney disease. There was no history of cutaneous melanoma. She reported no symptoms of hyper or hypothyroidism. In addition, she denied symptoms of dysphagia, shortness of breath, hoarseness, or other compressive symptoms. There was no family history of thyroid cancer or prior personal history of head or neck radiation. Home medications included alendronate, olmesartan, lorazepam, memantine, and vitamin B12. Her physical examination was only remarkable for a moderate thyroid struma with a 3 cm palpable firm nodule in the left lobe and a 3 cm nodule in the right lobe. Her thyroid stimulating hormone (TSH) level was normal at 1.76 mIU/L (reference range: 0.27–4.2 mIU/L), and her free thyroxine level was normal at 1.0 ng/dL (reference range: 0.9–1.7 ng/dL).

**Figure 2:**
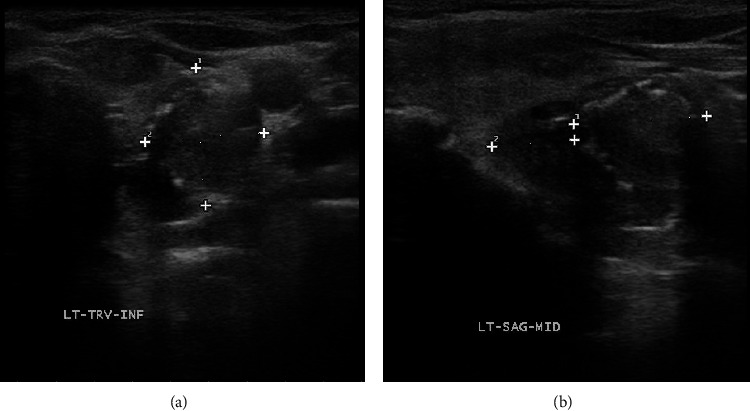
Transverse (a) and sagittal (b) view of the left lobe nodule on thyroid US. This nodule is a hypoechoic, solid, vascular lesion with inhomogeneous appearance, poorly defined border and coarse calcification.

**Figure 3:**
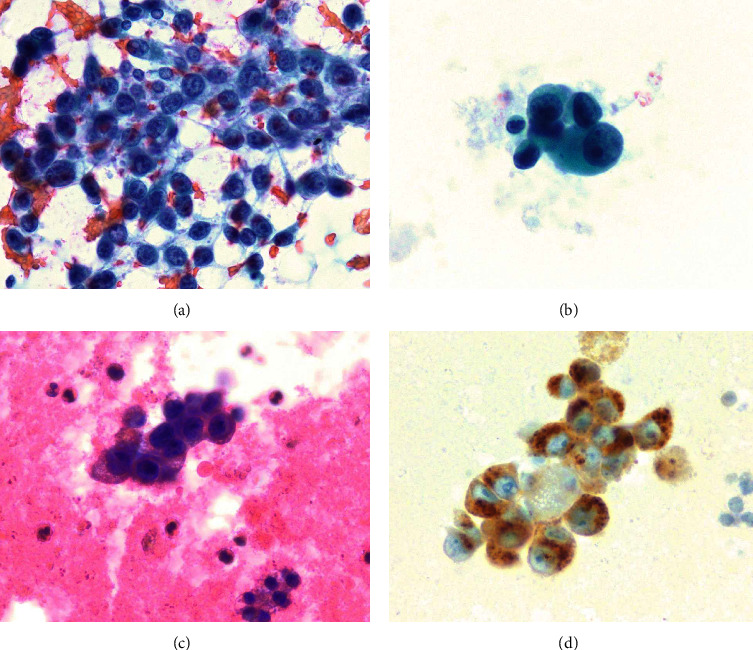
The thyroid fine needle aspirate (FNA) showed poorly cohesive epithelioid cells in a background of blood ((a) smear, papanicolaou stain, original magnification ×400). The tumor cells had abnormal chromatin content, prominent nucleoli, and dense cytoplasm ((b) thinprep, papanicolaou stain ×600). Same appearing tumor cells on cell block ((c) H&E stain, original magnification ×600). The tumor cells were positive for cytoplasmic melan-A stain ((d) immunohistochemical stain, original magnification ×600).

Ultrasonography of her thyroid demonstrated numerous vascular nodules in both lobes, and the largest nodule on the right and left lobes measured 3.4 × 2.4 × 2.1 cm and 3.4 × 1.9 × 2.5 cm, respectively. These TI-RAD 5 nodules were hypoechoic, solid, vascular lesions with inhomogeneous appearance and poorly defined borders. The left lobe dominant nodule also had coarse calcification (Figures 2(a) and 2(b)). Both were considered highly suspicious of malignancy based on their imaging features. The left dominant nodule had two ultrasonography-guided FNAs, and it was indeterminate the first time and nondiagnostic the second time. The ultrasound-guided FNA of the right dominant nodule was nondiagnostic twice but showed malignant cells the third time (Figures 3(a) and 3(c)). The atypical epithelioid cells were notable for overall large size with relatively preserved nuclear-to-cytoplasmic ratio, abnormal chromatin content, prominent nucleoli, and dense cytoplasm (Figure 3(b)). No melanin pigment is seen. Subsequent immunohistochemistry staining of the third FNA of the right thyroid nodule was positive for melanin A (Figure 3(d)), Mart-1, MSA, and SOX10 and negative for S100, pankeratin, TTF-1, and calcitonin. The overall cytologic features and immunoprofile were consistent with metastatic malignant melanoma. According to the thyroid metastases, a fluorodeoxyglucose (FDG)-positron emission tomography (PET) scan was performed for further staging, which demonstrated multiple areas of intense uptake in the brain, thyroid, liver, adrenal gland, axial, and appendicular skeleton supporting diffuse metastatic disease. Patient was referred to an oncologist but due to poor functional status, she elected not receiving any treatment and died within a year of diagnosis.

## 3. Discussion

Metastasis to the thyroid by uveal melanoma has been reported rarely in the past. The first case report on thyroid metastasis was a 72-year-old woman who was found to have thyroid metastasis before the diagnosis of uveal melanoma [14]. Another thyroid metastasis case developed 2 years after the diagnosis of uveal melanoma along with lung and adrenal metastases [15]. In our case, thyroid metastases were discovered 3 months after the diagnosis of uveal melanoma. Given the short interval, it is possible that the metastases were already present at the time of diagnosis, but a timely staging was not performed by the outside hospital initially. Alternatively, this may suggest the aggressiveness nature of the uveal melanoma observed in the present case. The diagnosis of thyroid metastases in our patient prompted further evaluation of systemic metastasis, and she was found to have multiple metastases and died within a year of diagnosis. Uveal melanoma metastasis typically develops within 15 years of initial diagnosis [16]. Once metastasis develops, 80% of patients succumb to it within 1 year [12].

Uveal melanoma often presents with visual symptoms or is discovered during routine examination as seen in this case. Host factors such as light eye color, fair skin color, or propensity to sunburn were associated with higher incidence of uveal melanoma [17]. Prognosis seems to be poorer in those with epithelioid cell type compared to those with spindle cell type [18]. Other prognostic factors included large tumor size, involvement of the ciliary body, increased age, extrascleral extension, lymphocyte infiltration, and increased mitotic activity [18]. Our case's advanced age, large tumor size, and epithelioid cell type clearly put her at high risk for poor outcome.

Cutaneous melanoma rarely metastasizes to the eye [19]. There were often a preceding history of cutaneous melanoma and prior multiple metastases to other organs before the discovery of ocular tumor. In addition, typically multiple flat metastases were found on fundoscopic exam [20]. This is different from our case as there was no history of cutaneous melanoma and there was only one large pigmented ocular tumor, classic for uveal melanoma. Lastly, cutaneous melanoma normally metastasizes first to local lymph node or subcutaneous tissue, and lung is the most common distant site of metastasis [19]. Despite showing diffuse metastases, our case did not have lymph nodes or lung metastasis. All these findings support the diagnosis of primary uveal melanoma.

In terms of diagnostic tests, thyroid ultrasound was the initial diagnostic modality which characterized the nodules in our case given the nodule was first found on physical examination. This route of discovery is quite different from the 2 prior case reports while they were found during a surveillance CT scan or incidentally identified during evaluation of a neighboring nonradioactive nodule [14, 15]. Interestingly, the nodules in prior studies were all much smaller as one measured 0.5 cm while another measured only 0.8 cm in contrast to the larger over than 3 cm nodules found in the current case [14, 15]. According to the ultrasonographic examination, similar to the prior study, the malignant nodules were hypoechoic, solid, vascular lesions with inhomogeneous appearance [15]. Hepatic metastasis is the most common site of metastasis; hepatic ultrasonography or other imaging examinations could have been very valuable if obtained at the time of diagnosis for staging purposes or while waiting for thyroid nodule biopsy results. If metastasis was suggested, then more tests would have been done earlier although it may not change the course of management in this particular patient given her age and comorbidities. FDG-PET is not a routine diagnostic test performed before thyroid surgery for patients with primary thyroid malignancy unless the tumor is undifferentiated or poorly differentiated. In contrast, PET/CT plays an important role in the staging of melanoma and has proven to be very valuable in this case, as it assesses the extent of the disease and helps predicate the prognosis. Newer diagnostic modalities, such as hybrid PET/contrast-enhanced CT or PET with new radiopharmaceuticals, could improve the potential value of this diagnostic tool in this field [21].

The diagnosis of metastasis to the thyroid on cytology could be difficult if there were inadequate or scant cellularity precluding further evaluation or if the metastasis were mistaken for follicular nodule or poorly differentiated thyroid cancer [3, 5]. In one study, the reported FNA accuracy rate ranged between 50 and 94.7% among different types of cancer, and 20% of cases of malignant melanoma were missed [4]. The diagnostic difficulty could be due to the atypical appearance of the cells and/or inadequate clinical information to alert the cytopathologist, the potential differential diagnosis of metastatic disease, or inadequate sampling. It is not easy to make a diagnosis even on frozen sections as metastases have been confused with medullary thyroid cancer, anaplastic thyroid cancer, follicular tumor, trabecular adenoma, or epithelial cells with atypia [3].

Unlike prior reported cases [14, 15], when the diagnosis was readily made on cytopathology, we have encountered great difficulty and needed to perform multiple FNA in achieving a definite diagnosis; this is likely due to the less classic cytopathology features observed in this case, namely, pure epithelioid morphology, lack of melanin pigment, and absence of S100 immunostaining. Both prior cases showed a mixture of spindle and epithelioid morphology as well as intracytoplasmic melanin pigment [14, 15]. Spindle cells are often a clue for uveal tract melanoma as they are unusual for cutaneous melanoma [14]. While in our case, the lack of spindle cells with morphology made uveal melanoma diagnosis difficult. Additionally, the absence of melanin pigment is uniquely seen in our case compared to the published reports, and this adds more diagnostic challenge as the existence of melanin pigment often points out to a diagnosis of melanoma [14, 15]. In terms of immunohistochemical staining, melanoma cells are generally positive for the first line melanoma marker such as S100 as reported in one prior case [15]. In our case, S100 staining was negative; hence, additional melanoma markers had to be used to support the diagnosis of melanoma in the presence of suspicious morphology. The diagnosis was finally made only after considering metastasis and obtaining an adequate sample for immunohistochemistry staining with antibodies against melanocyte differentiation antigens. Retrospectively reviewing the case, her FNA did show poorly cohesive cells (Figure 3(a)) which are not typical for thyroid neoplasm and should have raised concern for metastasis from other malignancies especially for melanoma given it is often dyshesive. Therefore, metastatic disease should always be considered in a patient with a history of cancer presenting with thyroid nodules, and a proper clinical history should be provided to the cytopathologist during the process of diagnosis.

There is no curative systemic treatment for the majority of patients with metastatic uveal melanoma although a durable response has been seen with ipilimumab use [10]. In a patient who developed systemic disease from choroidal melanoma, the thyroid metastasis was stable during ipilimumab treatment [15]. Our patient had poor functional status and could not receive any systemic therapy.

In conclusion, uveal melanoma is uncommon and metastasis to the thyroid is even rarer. Cytological diagnosis could be a challenge, but considering metastasis in the differential diagnosis and applying appropriate immunohistochemistry staining may facilitate correct diagnosis in a timely fashion.

## Data Availability

The data used to support the findings of this study are included within the article.
